# 金属有机骨架材料在色谱固定相构建及应用中的研究进展

**DOI:** 10.3724/SP.J.1123.2023.07029

**Published:** 2023-10-08

**Authors:** Meiting YAN, Wenwen LONG, Xueping TAO, Dan WANG, Zhining XIA, Qifeng FU

**Affiliations:** 1.西南医科大学药学院, 四川 泸州 646000; 1. School of Pharmacy, Southwest Medical University, Luzhou 646000, China; 2.重庆大学药学院, 重庆 401331; 2. School of Pharmaceutical Sciences, Chongqing University, Chongqing 401331, China

**Keywords:** 金属有机骨架, 色谱固定相, 分离, 综述, metal-organic frameworks (MOFs), chromatographic stationary phase, separation, review

## Abstract

金属有机骨架(MOFs)是由金属中心或团簇与有机配体组装而成的一类新型晶体多孔材料,具有比表面积大、孔隙率高、孔径均匀以及结构多样等优良特性,已被广泛应用于催化、吸附、传感、样品前处理以及色谱分离等领域。近年来MOFs在色谱分离领域的应用备受关注。与传统色谱固定相材料(如介孔二氧化硅、纳米粒子以及多孔层等)相比,MOFs具备灵活可调控的孔道尺寸和结构,能够实现对分子间相互作用的精确控制。此外,种类丰富多样的功能配体和拓扑结构拓宽了MOFs在分离领域的应用范围,有望实现更多类型复杂样品的分离分析。MOFs的这些独特优势使其非常适用于构建各类新型色谱固定相。迄今为止MOFs色谱固定相已展现出优异的分离效能,在色谱分离领域具有明显的优势和巨大的应用潜力。本文重点介绍了MOFs色谱固定相的构建方法及其在色谱分离应用中的最新研究进展,包括高效液相色谱(HPLC)、气相色谱(GC)以及毛细管电色谱(CEC)领域;针对现有的MOFs色谱固定相制备方法进行了归类总结,并简要探讨了各个方法的优缺点及发展方向;还总结了近年来MOFs色谱固定相的典型应用;最后,本文对MOFs色谱分离介质未来的研究重点及发展前景进行了展望,以期为先进MOFs色谱固定相的理性构建与应用提供参考。

## Abstract (en)

Metal-organic frameworks (MOFs) are a class of porous crystalline materials composed of metal centers or clusters assembled with organic ligands. These materials possess excellent properties, such as large surface areas, high porosities, uniform pore sizes, and diverse structures. Thus, MOFs have been widely applied in various fields, including catalysis, adsorption, sensing, sample pretreatment, and chromatographic separation. The applications of MOFs as stationary phases for chromatographic separation and analysis have attracted considerable attention from the research community in recent years. Compared with traditional chromatographic stationary phases, such as mesoporous silica, nanoparticles, and porous layers, MOFs possess flexible and tunable pore sizes and structures, thereby enabling precise control over their intermolecular interactions. Furthermore, the wide range of functional ligands and topologies of MOFs could potentially facilitate the separation and analysis of complex samples. These unique advantages render MOFs highly suitable for constructing novel chromatographic stationary phases.This article focuses primarily on the construction methods of MOFs as chromatographic stationary phases, and provides an overview of the latest research advancements in their applications in several chromatographic separation techniques such as high performance liquid chromatography (HPLC), gas chromatography (GC), and capillary electrochromatography (CEC). The existing methods for the preparation and construction of MOFs-based chromatographic stationary phases are classified and evaluated. The construction methods for MOFs as stationary phases for HPLC mainly include filling, precursor-doped polymerization, and post-modification. The construction methods for MOFs as stationary phases for GC predominantly include in situ growth, static coating, and dynamic coating. The stationary phases for CEC can be categorized into packed columns, monolithic columns, and open-tubular columns. Compared with monolithic and packed columns, open-tubular CEC (OT-CEC) offers numerous advantages, including a more flexible and convenient preparation method, enhanced compatibility with various separation media, and higher separation efficiency. Consequently, OT-CEC has emerged as an important method for investigating the preparation of stationary phases for CEC. Several methods such as physical adsorption, covalent attachment, and electrostatic interactions have been developed for the preparation and modification of MOFs-based CEC stationary phases, and extensive studies have been conducted to optimize the performance and applications of MOFs in OT-CEC. However, the existing methods for constructing MOFs-based chromatographic stationary phases present certain limitations. Therefore, the selection of the appropriate MOFs, optimization of their preparation methods, and examination of their performance in different separation modes have become the focus of intensive research.This review also summarizes the different analytical targets (e. g., chiral small molecules, biomacromolecules, and nonchiral molecules) and corresponding separation effects achieved using various MOFs-based chromatographic stationary phases. Finally, future studies focusing on the development of MOFs as chromatographic separation media are discussed. Overall, this review provides a valuable reference for the rational construction and practical applications of advanced MOFs-based chromatographic stationary phases.

色谱技术以其能够对各类样品进行高效分离分析而被广泛应用于化学分析、生命科学、环境科学等领域。然而,随着各类新型复杂样品体系的不断涌现,高效色谱分离分析的新需求日益迫切,因此色谱技术的分离效能亟需进一步提升。作为色谱分离技术的重要组成部分,固定相的性能对于实现高效色谱分离分析至关重要^[1]^。

金属有机骨架(metal-organic frameworks, MOFs)是一类由金属离子或金属簇与有机配体有序组装而成的新型多孔晶体材料。MOFs具有制备方法简便且温和、比表面积大、孔隙率高、热稳定性好、孔径及活性位点灵活可调等优异特性,在色谱分离领域受到了研究者们的广泛关注^[2,3]^。由于其独特的结构和特性,MOFs已成为一类高性能色谱分离固定相,在复杂样品分离分析领域具有显著的应用潜力。本文主要聚焦于近年来在高效液相色谱(HPLC)、气相色谱(GC)和毛细管电色谱(CEC)等领域的MOFs色谱固定相构建方法,并对不同制备方法的特点进行了探讨。此外,还综述了MOFs固定相在色谱分离中的最新应用情况。

## 1 MOFs色谱固定相的构建方法

### 1.1 MOFs高效液相色谱固定相的制备

MOFs材料作为新兴的先进分离介质,在色谱固定相领域已展现出巨大的应用价值。按照构建方式的不同,MOFs液相色谱固定相主要可分为填充柱和整体柱两种类型。填充型HPLC固定相的制备方法主要有MOFs材料直接装填法和MOFs基复合材料装填法。Maes等^[4]^采用干填充法,直接将MIL-47作为填料填充至不锈钢柱中,在色谱分离实验中成功实现了乙苯和苯乙烯的基线分离。研究结果还表明,MIL-47固定相在分离过程中表现出分子筛效应和呼吸效应。严秀平课题组^[5]^采用匀浆法填充MIL-53(Al)柱,并借助二元流动相HPLC方法,成功实现了对二甲苯、二氯苯、氯甲苯和硝基苯酚异构体的基线分离,同时展现了高柱效的优势。然而,直接将MOFs颗粒作为固定相时,常常面临背压过大、填充不均匀和各向异性等问题,从而限制了MOFs晶体作为HPLC固定相的应用。

2010年,均匀球形MOFs基复合材料作为HPLC固定相的策略被提出,一定程度上解决了MOFs直接作为固定相填料所带来的高背压、低柱效等问题,已被广泛用于HPLC分离^[6]^。Ehrling等^[7]^采用逐层法制备了多种微孔MOFs@SiO_2_核-壳复合微球并将其作为HPLC固定相,在正相模式下成功实现了C8异构体、二氯苯异构体、苯乙烯和乙苯的高效分离。此外,液相外延(LPE)技术可用于在功能化硅球表面生长出具有高结晶质量、可控取向和厚度的均匀MOF膜,已成为合成均匀MOFs复合填料的重要方法之一^[8]^。然而,LPE和逐层法(LBL)工艺步骤较为繁琐,实用性受限。丁明玉课题组^[9]^提出了一种简单易行的一锅合成法,通过调节反应参数即可合成具有不同负载量的SiO_2_@UiO-66核-壳复合微球,成功应用于HPLC分离二甲苯异构体和乙苯等有机小分子化合物(图1)。尽管SiO_2_是目前最常用的MOFs负载球形颗粒,但MOFs@SiO_2_核-壳复合微球仍面临不均匀团聚以及材料兼容性差等问题。因此,需进一步探索和开发新型MOFs复合填料,更好地控制MOFs颗粒生长的均匀性和厚度,进而拓展MOFs作为高性能HPLC固定相的应用价值。

**图 1:**
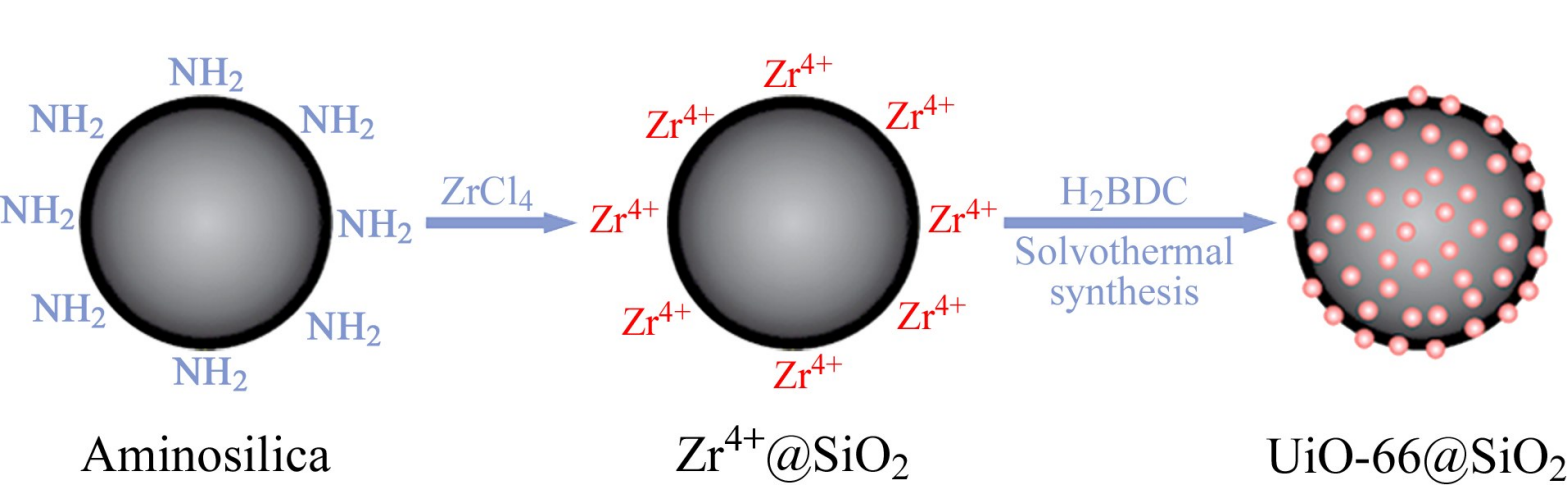
一锅合成法制备SiO_2_@UiO-66核-壳复合微球^[9]^

相比填充柱,MOFs整体柱具有制备过程简单、稳定性良好、孔隙丰富及易功能化等优势,常用于毛细管液相色谱(CLC)中。整体柱的制备方法主要有前驱体掺杂聚合法和后修饰改性法。Yang等^[10]^利用逐层自组装后修饰策略,在羧基官能化聚(甲基丙烯酸-共-二甲基丙烯酸乙二酯)(poly(MAA-co-EDMA))整体柱内原位生长HKUST-1,并在反相模式下对二甲苯、苯二酚、乙苯和苯乙烯等小分子化合物实现了高效分离,且分离效能随着HKUST-1载量的增加而提升。Pérez-Cejuela等^[11]^基于NH_2_-MIL-101(Al)制备了MOF改性聚甲基丙烯酸缩水甘油酯-二甲基丙烯酸乙二醇酯(poly(GMA-co-EDMA))整体柱,并实现了多环芳烃和非甾体抗炎药的基线分离。作者对比了前驱体掺杂聚合和后修饰等不同方法制备的HKUST-1整体柱性能,发现后修饰方法会导致MOF过度堆积从而阻塞孔隙,影响MOFs对色谱柱的均匀修饰。尽管整体柱具备上述优点,但由于聚合过程的不可控性以及内部结构不均匀等问题,MOFs在整体柱HPLC中的应用仍受到限制。因此,有必要开发聚合路径简单可控、结构稳定均匀的MOFs整体柱以提高其性能。

Ding等^[12]^通过交联分子(CLMs)共价组装方法制备了UiO-66/NH-MA@CLM杂化整体毛细管柱,通过MOFs本身以及MOFs和聚合物之间的交联形成可调的分级孔结构,在反相CLC模式下同时分离低相对分子质量化合物和聚合物并具有良好的重现性(图2)。张维冰课题组^[13]^利用后修饰方法在刷状聚甲基丙烯酸缩水甘油酯(pGMA)涂层毛细管(25 μm内径)内壁表面偶联NH_2_-UiO-66纳米颗粒。所获得的NH_2_-UiO-66改性pGMA涂层开管毛细管柱具有较高的NH_2_-UiO-66负载量及良好的柱渗透性,可成功用于芳香烃、烷基苯、苯酚等小分子化合物的液相色谱分离,分离效率可达108462板/米。此后,该课题组^[14]^通过前驱体掺杂聚合策略成功制备了IRMOF-3@vancomycin-pMSA多孔层开管(PLOT)毛细管柱,在亲水作用色谱(HILIC)模式下,对中性、酸性和碱性化合物进行了分离分析,并进一步实现了牛血清白蛋白(BSA)胰酶消化液的梯度洗脱高效分离。与聚合物整体柱相比,PLOT柱的多孔聚合物薄层厚度和孔结构易受到聚合反应条件影响,须对聚合速率等进行严格控制,才能取得较好的分离重现性。相比常规HPLC分离模式,CLC使用内径较小的毛细管柱作为分离通道,使色谱分析更趋于微型化和绿色化。MOFs分离介质与CLC技术相结合,有望获得更出色的动力学和分离性能,在色谱领域具有巨大的应用潜力。

**图 2:**
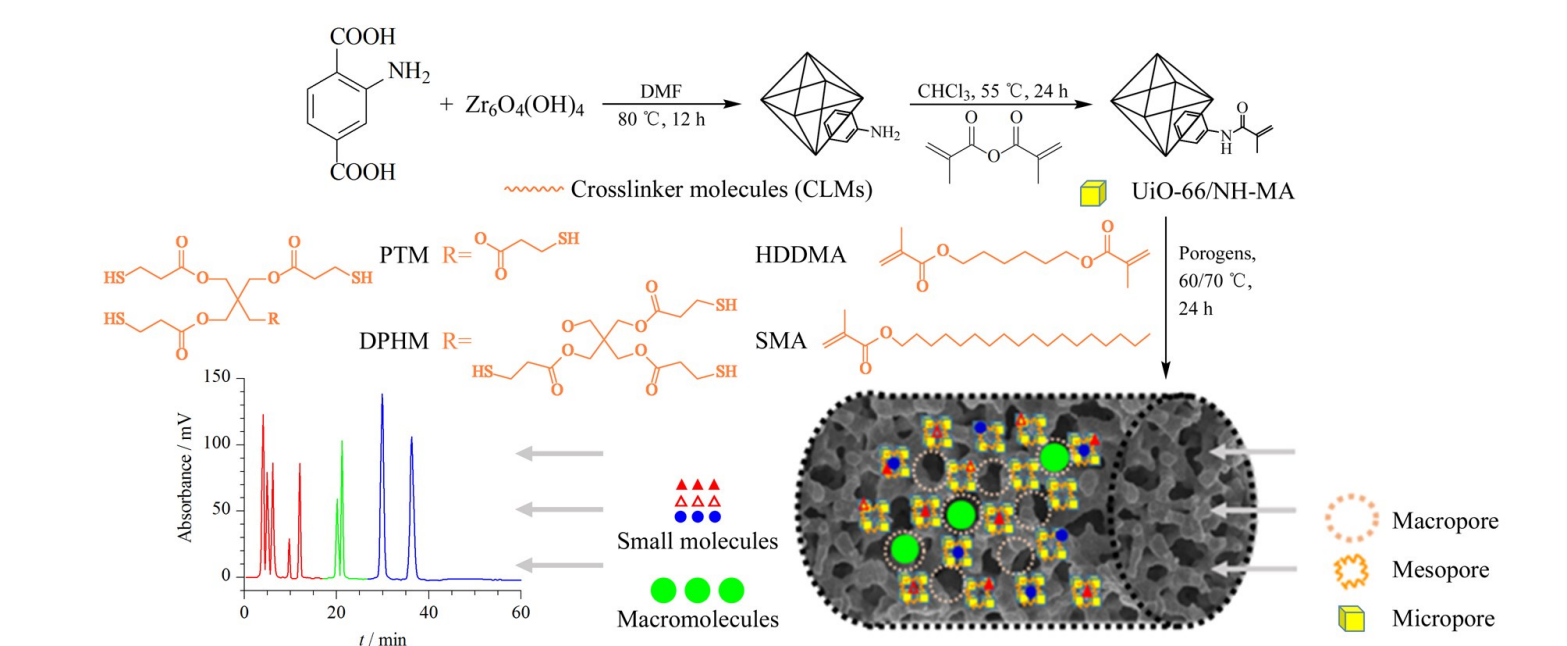
UiO-66/NH-MA@CLM杂化整体柱的制备与应用^[12]^

### 1.2 MOFs气相色谱固定相的制备

MOFs材料作为高效GC分离介质,已在挥发性复杂样品的分离分析中取得了显著的研究进展。MOFs材料可直接填充在玻璃管、不锈钢柱等载体中,用于GC分离分析。然而,不均匀的填料颗粒经常导致重现性不佳、峰形展宽、柱效下降等问题,显著影响GC填充柱的分离效能和实用性^[15]^。与填充柱相比,MOFs掺杂聚合物整体柱的渗透性更好,制备方法也较为简便,在GC固定相领域有着一定的应用价值。Yusuf等^[16]^通过前驱体掺杂策略,制备了ZIF-8掺杂聚甲基丙烯酸(BuMA-co-EDMA)整体柱,成功用于线性烷烃及油漆稀释剂的GC快速分离分析。

相比于MOFs掺杂整体柱,MOFs涂层开管(OT)模式的GC分离柱效较高,且制备方法更加简便高效,因此近年来受到广泛关注和应用。MOFs涂层GC固定相可通过原位生长法和后修饰法进行制备^[17,18]^。原位生长法是一种将MOFs晶体直接生长在毛细管内壁上的方法。Münch等^[19]^采用循环层沉积方法在毛细管内壁原位生长了HKUST-1涂层GC固定相。该方法与传统层层自组装涂层方法的主要区别是通过氩气气流直接吹掉单层反应后残留的前驱体溶液,有助于前驱体液膜的保留及MOFs涂层的高效生长。所制得的HKUST-1涂层柱不仅具有较高的涂层质量,而且展现出良好的GC分离性能。Wu等^[20]^通过Zn(II)与羧基的配位反应,成功将ZIF-90晶体原位生长于毛细管内壁上,所制得的ZIF-90改性毛细管GC柱实现了对多种极性化合物(包括酮类、醇类、醛类等)的有效分离(图3)。

**图 3:**
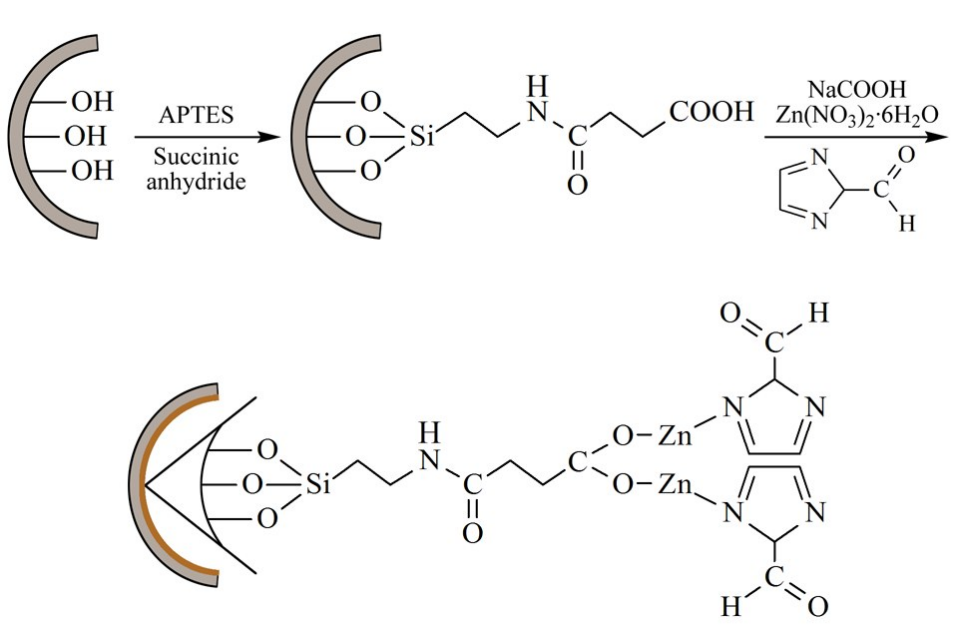
ZIF-90键合毛细管柱的制备示意图^[20]^

相比于合成过程耗时较长且可控性较差的柱内原位生长策略,静态和动态涂覆等后修饰涂层方法适用于更多种类MOFs改性GC毛细管柱的高效制备,并受到更多研究者的关注和应用。静态涂覆MOFs改性毛细管柱通常是将MOFs分散液静置在毛细管内,使其自发涂覆在管壁上,最终形成均匀的MOFs涂层^[21]^。Patel等^[22]^将柱外预先合成的ZIF-8晶体分散在离子液体中,并采用静态涂覆法制备ZIF-8改性毛细管柱用于气相色谱的分离。静态涂覆法制备的MOFs涂层GC毛细管柱通常具有良好的分离性能,但由于操作复杂且制备时间较长,还需进一步研究和改进,以提高实用性和分离效率。

动态涂覆法是在惰性气体气流的作用下,采用固定相溶液持续冲洗毛细管柱,然后使管内溶剂挥发,最后分离介质覆盖在毛细管内壁形成一层固定相膜^[23]^。2010年,严秀平课题组^[24]^首次报道了通过动态涂覆法制备MOFs(MIL-101)改性GC毛细管柱,仅需1.6 min即可实现对二甲苯的高效基线分离。Meng等^[25]^采用动态涂覆法制备了3种具有不同配体的锆基MOFs(NU-1000、PCN-608和PCN-222)改性毛细管柱,并以烷烃、烷烃异构体和取代苯异构体为例,研究了MOFs固定相颗粒大小对GC分离性能的影响。Tian等^[26]^提出了一种新的动态涂覆GC毛细管柱制备方法,即利用184 silicone作为MAF-5的保护薄膜,显著提升了MAF-5涂层的稳定性和完整性,并将其成功用于直链烷烃、多环芳烃以及有机氯农药等分析物的快速分离分析。尽管动态涂覆法操作简便、涂覆速度快,且制备的色谱柱通常具有较高的分离能力,但该方法仍存在一定的局限性,例如液膜不均匀和重复性较差。Wang等^[27]^提出一种聚硅氧烷基辅助制备晶体海绵(MOFs类似物)涂层手性GC固定相的新方法,即在聚硅氧烷(polysiloxane, PSO)OV-1701的辅助作用下,将手性晶体海绵(chiral crystalline sponges, CCS-3S)牢固黏附在毛细管内壁上。该PSO/CCS-3S涂层柱表现出更高的分离选择性,并且能够在更广泛的对映体分离范围内发挥优异的手性分离能力(图4)。

**图 4:**
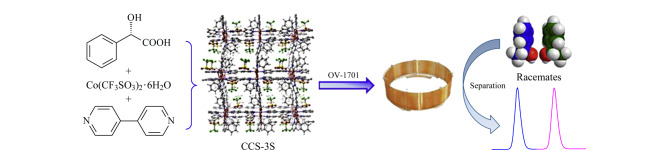
OV-1701辅助制备PSO/CCS-3S涂层毛细管柱的示意图^[27]^

### 1.3 MOFs电色谱固定相的制备

依据固定相的形式,CEC可分为填充柱(PC-CEC)、整体柱(MC-CEC)和开管柱(OT-CEC)三大类。PC-CEC通常是将各类分离介质填充于毛细管内,再经过两端柱塞烧结来制备的。Fei等^[28]^采用加压填充法将手性MOF [In_3_O(obb)_3_(HCO_2_)(H_2_O)]晶体直接装填于毛细管内,在CEC模式下,实现了克仑特罗、氢化安息香等手性对映体以及硝基酚同分异构体的快速有效分离。但PC-CEC的分离性能易受填料粒径分布、形貌差异等因素的影响,且制备过程较为复杂,目前已逐渐被MC-CEC和OT-CEC所取代。Ding等^[29]^采用层层自组装原理在聚合物整体柱内原位生长ZIF-8晶体涂层,再利用戊二醛交联原理在ZIF-8晶体表面键合胃蛋白酶分子,所制得的pepsin-ZIF-8-poly(GMA-co-EDMA)整体柱对羟氯喹、氯喹、羟嗪等手性药物的CEC拆分效果显著。Miao等^[30]^采用相似的策略成功制备了pepsin@MOF-5@poly(GMA-co-EDMA)整体毛细管柱,并利用其成功实现了奈福泮、克仑特罗及氯苯那敏等6种碱性手性药物的CEC手性拆分。Zhang等^[31]^将镧系金属MOF(NKU-1)分散至预聚合混合物中,并将该混合溶液通入毛细管内,采用前驱体掺杂MOFs原位聚合策略成功制备了NKU-1-poly (BMA-co-EDMA)整体柱,实现了对烷基苯、多环芳烃等多类化合物的高效CEC分离。MOFs改性MC-CEC的固定相载量较大,易于获得较高的分离选择性,但该模式普遍存在柱压较高、重现性较差、毛细管易堵塞等问题,一定程度上限制了MC-CEC的应用。

相比于填充柱和整体柱,OT-CEC的固定相制备方法更加灵活多样,且与新型色谱分离介质更加兼容,已逐渐发展成为一类主流的CEC固定相制备方式。然而,开管模式的固定相负载量有限,导致OT-CEC柱的容量较小且分离选择性受限。因此,迫切需要开发具有高负载量和更大柱容量的新型OT-CEC固定相。MOFs具备改善固定相传质性能和增加作用位点数量的双重优势,在OT-CEC领域已得到广泛应用。迄今为止,MOFs用于构建OT-CEC固定相的制备方法主要包括物理涂覆法、化学键合法和原位生长法等。

物理涂覆法是一种后修饰固定方法,依靠范德华力、氢键或静电作用等非共价键相互作用力将MOFs涂层覆盖在毛细管内壁上。该类方法制备过程简便,易于实现,适用于多数MOFs材料。Wang等^[32]^利用静电吸附将BSA与ZIF-8结合修饰于毛细管内壁,在CEC模式下成功分离了多种同系物和异构体。Zhang等^[33]^采用相似的方法制备了组氨酸修饰的ZIF-8涂层毛细管柱,成功分离了3种外消旋碱性药物的对映体。研究发现,该手性MOFs能够与*β*-环糊精(*β*-CD)起到协同作用,与不含His-ZIF-8的CM-*β*-CD毛细管柱相比,His-ZIF-8柱的分离选择性显著提升。硅酸钠作为一种良好的无机黏附剂,可显著增强MOFs晶体与毛细管内壁的结合能力。Fei等^[34]^利用硅酸钠的黏附作用将手性MOFs材料[Zn_2_(D-Cam)_2_(4,4'-bpy)]*_n_*均匀、致密地固定于毛细管内表面,并利用该手性涂层柱实现了黄酮和吡喹酮的CEC基线分离。此外,陈子林课题组^[35]^利用聚多巴胺(PDA)的多活性基团和强黏附特性,将预先合成的*γ*-CD-MOF(Cu-SD)涂覆在PDA改性毛细管内,得到的Cu-SD改性柱对丹酰化氨基酸对映体具有良好的手性分离效果。然而,物理涂覆方法所得MOFs改性OT-CEC柱的涂层结合力不强,易脱落且化学稳定性欠佳。

化学键合法主要通过使用硅烷偶联剂等连接剂,基于共价作用将MOFs固定在毛细管内壁。相较于物理涂覆法,化学键合法具有更高的固定强度,可以在一定程度上改善MOFs涂层的稳定性。Ye课题组^[36]^利用氨丙基三乙氧基硅烷(APTES)所携带氨基与MOFs晶体表面不饱和配位羧基基团间的酰胺缩合反应,首先制备得到APTES改性Mn(cam)(bpy),再利用硅烷偶联原理将该APTES改性MOFs晶体共价键合于毛细管内壁,进而将该Mn(cam)(bpy)涂层柱成功用于多种磺胺类化合物的CEC分离分析(图5)。此外,Ma等^[37]^通过甲基丙烯酸缩水甘油酯共聚物与组氨酸改性ZIF-67在毛细管内表面的化学键合,在常温条件下制备了组氨酸改性ZIF-67毛细管电色谱固定相。Sun等^[38]^采用共价交联策略制备了一种脂肪酶与MIL-100(Fe)相结合的复合材料,再使用3-三甲氧基丙基甲基丙烯酸酯(*γ*-MAPS)作为交联剂将该复合材料固定在毛细管内壁,通过CEC模式实现了多类对映体化合物的有效分离。化学键合法虽然可有效提高毛细管柱内壁涂层的稳定性,但由于其制备过程复杂且需要特定反应活性的官能团,仍存在一定的局限性。

**图 5:**
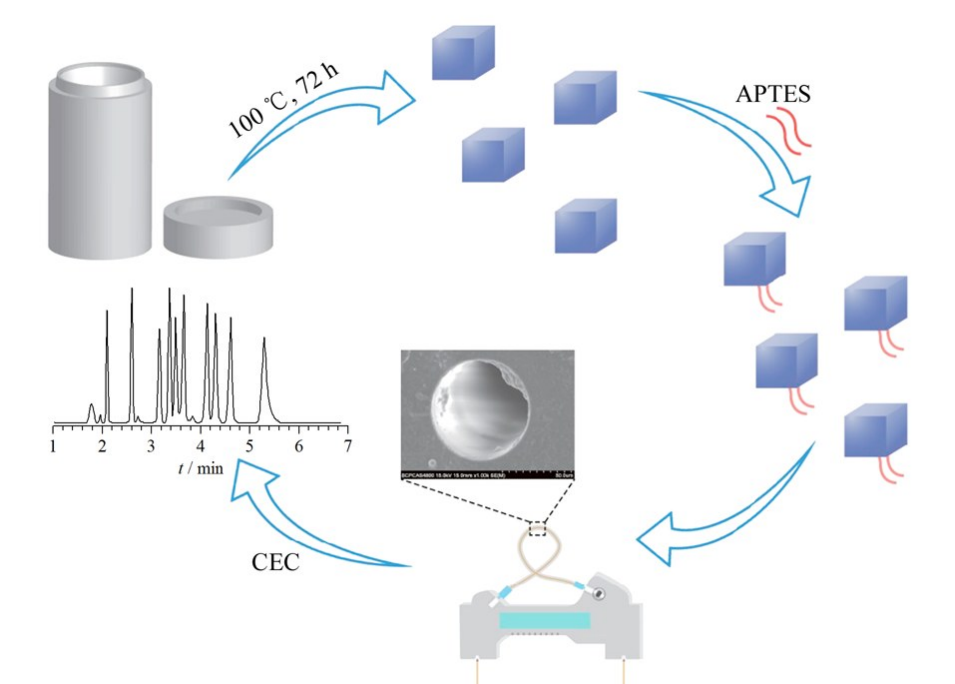
Mn(cam)(bpy)涂层柱的制备及应用示意图^[36]^

由于流动相对管壁的剪切力较强,相比于开管MOFs涂层GC柱,OT-CEC模式对MOFs固定相的涂层牢固性有着更高要求。与前述后修饰方法不同,原位生长法直接在毛细管内壁表面进行MOFs成核生长,有助于显著提升MOFs涂层与管壁的结合能力。Sun等^[39]^采用原位生长策略成功制备了L-组氨酸(L-His)改性MIL-53涂层柱。该方法首先将APTES、戊二醛以及高锰酸钾溶液依次通入毛细管中,得到羧基改性柱,然后通入MOFs前驱体溶液,在一定条件下于管壁界面原位生长NH_2_-MIL-53晶体,再利用酰胺缩合反应对所得MOFs涂层进行L-His改性,所得L-His-NH-MIL-53改性CEC柱对部分外消旋药物展现出较强的手性拆分能力。Zheng等^[40]^基于相似的制备策略,首先在毛细管壁原位生长NH_2_-MIL-53,再对其进行环糊精后修饰,所制得的Cyclodextrin-NH-MIL-53涂层柱对手性氨基酸呈现较好的手性拆分能力。本研究组受半胱氨酸(Cys)可有效诱导加速MOFs晶体成核的启发,发展了一种高效、通用的MOFs涂层固定相制备方法,即固定化半胱氨酸诱导原位生长(ICISG)^[41]^,可用于多种MOFs涂层毛细管固定相的高效制备(图6)。以4种由不同金属离子(Zn^2+^、Cu^2+^、Fe^3+^、Zr^4+^)组成的MOFs为例,采用ICISG策略,ZIF-8等MOFs晶体可快速生长于Cys改性毛细管内壁上,中性、酸性和碱性化合物在4种MOFs涂层柱上均实现了高效CEC基线分离。基于该ICISG策略,我们进一步制备了具有均一介孔结构的mesoMOF-1涂层柱^[42]^,并用于高效CEC分离分析。相较于HKUST-1涂层柱,mesoMOF-1涂层柱能更加有效地平衡各类分析物自身的动力学扩散及与介孔孔道间的热力学相互作用,成功实现了7种不同分子尺寸化合物的高效分离分析,理论塔板数高达1.4×10^5^板/米。此外,Li等^[43]^基于前驱体掺杂聚合原理,将CAU-1掺入聚甲基丙烯酸甲酯(PMMA)前驱体溶液中,在管壁表面原位聚合成功制得CAU-1@PMMA涂层PLOT柱,进而对芳香酸类化合物实现了更高柱效的CEC分离分析。尽管原位生长方法在MOFs涂层OT-CEC固定相开发中已展现出巨大的应用价值,但该策略在涂层方法学适用性和重现性等方面仍存在较大缺陷。进一步探索和发展简便、普适性强、结晶性优异且具有良好重现性的MOFs涂层OT-CEC固定相构建新方法具有重要意义。

**图 6:**
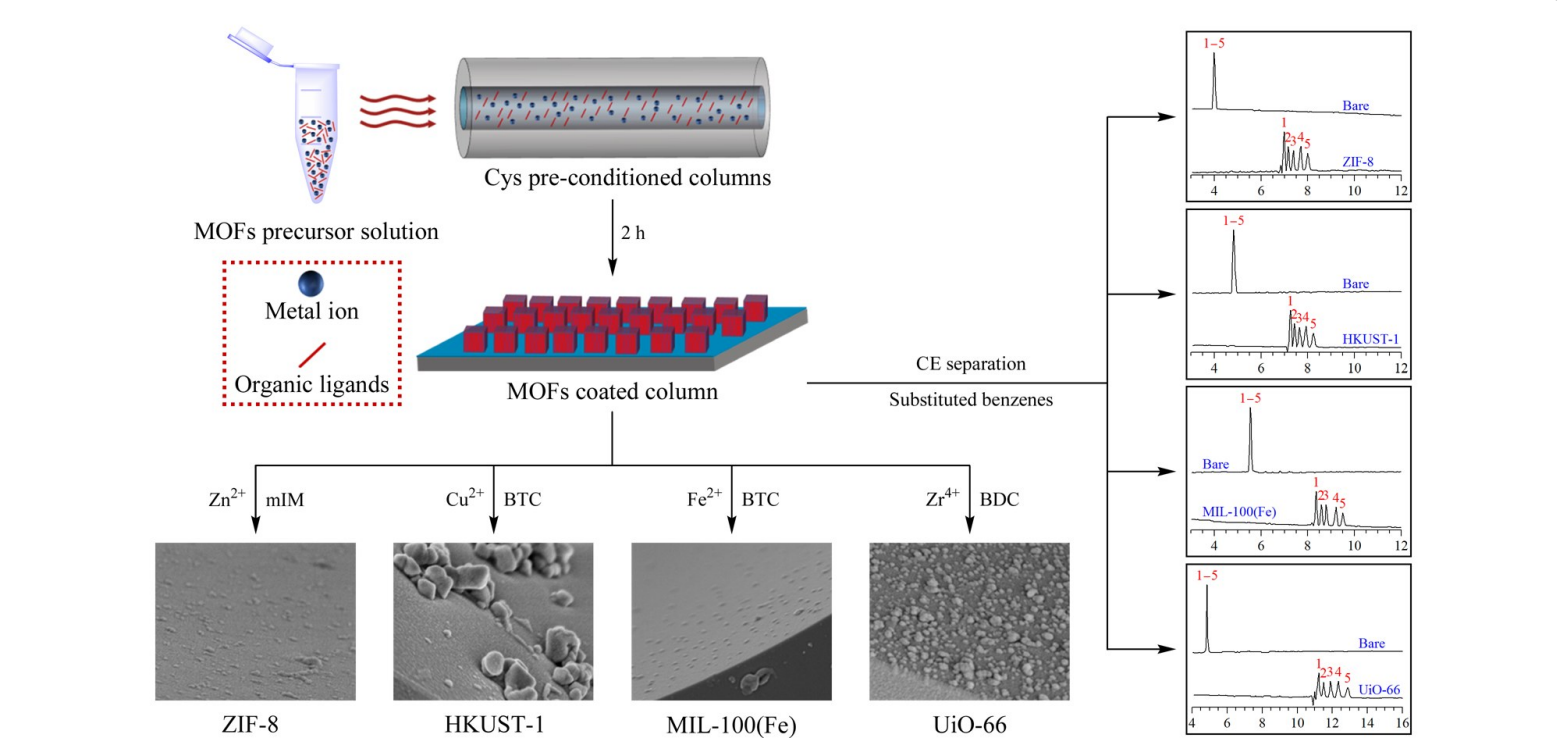
ICISG策略用于制备MOFs涂层柱^[41]^

## 2 MOFs色谱固定相的应用

MOFs作为一类新型色谱分离介质,已在HPLC、CEC、GC等色谱分离技术中得到广泛应用。由于GC分离过程所采用的温度较高,因此要求MOFs气相色谱固定相具有良好的热稳定性。HPLC和CEC分离则涉及大量流动相洗脱过程,因此要求MOFs液相色谱和电色谱固定相具有优良的溶剂稳定性。目前,已有许多种类的高稳定性MOFs分离介质被成功用于各类复杂样品的HPLC、CEC、GC分离分析。

### 2.1 MOFs液相色谱固定相的分析物类型

#### 2.1.1 手性化合物

众所周知,手性分离在生物医药领域具有重要意义。MOFs以其丰富的手性拓扑结构、可调节的孔道尺寸、多样化手性识别位点等诸多独特优势,在手性分离分析领域得到了广泛关注。Padmanaban等^[44]^制备了手性修饰的UMCM-1(Chir-UMCM-1),首次利用手性MOFs作为固定相进行HPLC手性分离。Tanaka等^[45]^通过手性有机连接体和金属离子配位合成了新型均一手性(*R*)-CuMOF-35。对12种外消旋体的拆分效果表明,此类均一手性MOFs固定相可用于高效手性识别与分离。Yu等^[46]^利用一锅合成方法制备了一种手性D-His-ZIF-8@SiO_2_核-壳微球复合材料,用于醇、酚、胺、酮和有机酸等18种外消旋化合物的HPLC手性分离,展现出良好的手性拆分能力。

#### 2.1.2 非手性化合物

除了手性化合物之外,MOFs固定相在非手性化合物的HPLC分离分析领域也获得了广泛应用。Ding等^[12]^利用交联分子共价组装方法制备了具有分级孔结构的UiO-66/NH-MA@CLM杂化毛细管整体柱,其能够通过尺寸筛分及静电作用等多重机制同时分离小分子化合物和多肽、蛋白质等大分子,并表现出良好的重现性。Qu等^[47]^通过将MOFs薄层生长在SiO_2_大孔表面,成功制备了具有树枝状孔道结构的SiO_2_@dSiO_2_-ZIF-8核-壳复合微球色谱柱,并应用于碱性(苯胺衍生物)和酸性(苯酚衍生物)分析物的分离分析。Si等^[48]^制备了新型MOF-808@SiO_2_核-壳结构填料,并将其用作HILIC固定相,对磺酰胺、生物碱、核苷、核碱、抗生素和氨基酸等亲水性分析物展现出更好的分离选择性和稳定性。此外,MOFs也常用作CLC固定相,用于多肽、蛋白质和核苷酸等生物大分子的分离分析。张维冰课题组^[14]^制备的IRMOF-3@vancomycin-pMSA多孔层开管毛细管柱,成功实现了BSA胰酶消化液的梯度洗脱CLC高效分离。表1总结了MOFs液相色谱固定相的主要类型及分析对象。

**表 1:** MOFs固定相在HPLC中的典型应用

Material	Analytes	Type	Form	Ref.
MIL-47	ethylbenzene and styrene	packed	dry-packed	[4]
MIL-53(Al)	xylene, dichlorobenzene, chlorotoluene and nitrophenol isomers	packed	slurry-packed	[5]
HKUST-1-poly(MAA-co-EDMA)	benzenediols, xylenes, ethylbenzene and styrene	monolithic	post-modification	[10]
NH_2_-MIL-101-(GMA-co-EDMA)	polycyclic aromatic hydrocarbons and non-steroidal anti- inflammatory drugs	monolithic	in-situ polymerization post-modification	[11]
NH_2_-UiO-66-modified pGMA	aromatic hydrocarbons, alkylbenzenes, phenols, anilines and flavonoids	OT-CLC	post-modification	[13]
IRMOF-3@vancomycin-pMSA	neutral compounds, aromatic acids, phenols and bovine serum albumin pancrease digestion fluid	OT-CLC	in-situ polymerization	[14]
(R)-CuMOF-35	secondary alcohols, sulfoxides, lactones and other racemes	packed	composites-packed	[45]
D-His-ZIF-8@SiO_2_	alcohols, phenols, amines, ketones and organic acids racemes	packed	composites-packed	[46
SiO_2_@dSiO_2_-ZIF-8	xylene isomers, basic and acidic compounds	packed	composites-packed	[47]
MOF-808@SiO_2_	antibiotics, carbohydrates and other hydrophilic substances	packed	composites-packed	[48]
ZIF-8-PEI-CA	troger bases racemic compounds	packed	slurry-packed	[49]
Zr_6_O_4_(OH)_8_(H_2_O)_4_(L)_2_	amino acid derivatives and drugs	packed	composites-packed	[50]
MIL-101@c-PANI	alcohol, ketones, esters, organic acids and amines racemes	packed	composites-packed	[51]
Co-MOF-74-L-Tyr	alcohol and phenolic racemes	packed	slurry-packed	[52]
UiO-66-DATA(DBTA)@SiO_2_	small molecule basic alpha-amino acid enantiomers	packed	composites-packed	[53]
Amino acids modified MIL-101	RS-ibuprofen, RS-mandelic acid and RS-1-phenylethanol	packed	dry-packed	[54]
UiO-67@SiO_2_	polycyclic aromatic hydrocarbons and thiourea compounds	packed	composites-packed	[55]
MOF-235@PEG@silica	alkaloids, nucleosides and nucleobases and sulfonamides	packed	composites-packed	[56]
MIL-53(Al)	alkyl benzene, xylene, ketones, phenols and alkaloids	packed	slurry-packed	[57]
SiO_2_@dSiO_2_-UiO-66	xylene isomers, aromatics, biomolecules and acid	packed	composites-packed	[58]
[Cu(S-mal)(bpe)]_n_	alcohols, acids, ketones and phenols racemic compounds	packed	slurry-packed	[59]

OT-CLC: open-tubular capillary liquid chromatography.

### 2.2 MOFs气相色谱固定相的分析物类型

#### 2.2.1 手性化合物

与HPLC不同,气相色谱在研究固定相与分析物之间的作用时,不受流动相溶剂的影响,其分离结果能直接反映固定相与分析物之间相互作用的强度。Sharafutdinova等^[60]^制备了[{C $u_{12}^{1}$ (trz)_8_}·4 Cl·8H_2_O]*_n_*改性手性毛细管柱,并将其用于多种手性化合物的GC手性分离分析。袁黎明课题组^[61]^将柱外合成的均一手性MOFs材料Co-L-GG动态涂覆到毛细管柱内壁上,用于GC手性分离。该Co-L-GG涂层手性GC柱可在7 min内成功分离烷烃衍生物、烯类化合物、酮和醇等手性化合物,表现出优异的手性分离效能。

#### 2.2.2 非手性化合物

烷烃是石油和化学工业中的重要原材料,目前已有大量基于GC技术对烷烃进行分离分析的相关报道。Read等^[62]^首次将HKUST-1和ZIF-8等MOFs用作一维和二维微芯片气相色谱(μGC和μGC×μGC)固定相,并成功实现了高挥发性轻质烷烃的快速分离。Wang等^[63]^成功合成了一系列以四异位羧酸酯连接剂构建的高度稳定的MOFs,并利用GC模式实现了C5~C6烷烃异构体的高效分离,展现出卓越的分离性能。这些研究结果表明,不同结构类型的MOF气相色谱柱在不同烃类分离过程中具有各自的优势。此外,通过改变MOFs的堆积方式也可以实现化合物的高效GC分离。Tao等^[64]^制备了扭曲和非扭曲堆积的超薄二维Zr-BTB-FA纳米片,并将其作为固定相应用于取代苯异构体的GC分离。结果显示,相比于扭曲堆积MOFs涂层柱,非扭曲Zr-BTB-FA纳米片涂层柱的选择性和分离效能得到显著提升。Tang等^[65]^通过不同主客体相互作用的诱导机制,合成了不同堆积模式的Zr基苯骨架纳米片。非扭曲有序纳米孔结构固定相对苯衍生物异构体展现出优异的GC分离效果,显著优于扭曲杂乱堆叠纳米片固定相,揭示出MOF材料堆积方式的重要性。综上所述,MOFs在挥发性复杂样品的GC分离分析中显示出令人瞩目的应用潜力。表2总结了MOFs气相色谱固定相的主要类型及分析对象。

**表 2:** MOFs固定相在GC中的典型应用

Materials	Analytes	Type	Form	Ref.
ZIF-8-butyl methacrylate monoliths	linear alkanes and paint thinners	monolithic	in situ polymerization	[16]
Zn_2_ (bdc)(L-lac)	diphenyl aromatic hydrocarbons derivatives	OT	static coating method	[18]
ZIF-90	alkane isomers	OT	in situ growth	[20]
Co-L-GG	halocarbons, ketones, esters, epoxides, alcohols	OT	dynamic coating	[61]
HKUST-1, ZIF-8	light-chain hydrocarbon mixtures (C1-C4)	OT	layer by layer deposition	[62]
[Cu(sala)]_n_	racemic, alkanes, alcohols,	OT	dynamic coating	[66]
[Cd(LTP)_2_]_n_	racemic compounds, mixed alcohols, α,β-ionone	OT	dynamic coating	[67]
SIFSIX-1, SIFSIX-3	alkane isomers, benzene series	OT	dynamic coating	[68]
ZIF-8	cyclohexane, n-hexane, n-heptane, n-octane, n-decane	OT	dynamic coating	[69]
HKUST-1	methane, ethane, propane, n-butane	OT	dynamic coating	[70]
MOF-5	xylene isomers	OT	in situ growth	[71]
MIL-101(Al)-NH_2_-Xs	ethyl toluene isomer	OT	covalently bonding	[72]
Cd(D-Cam)(tmdpy)	aromatic and linear alkane derivatives	OT	dynamic coating	[73]
UiO-66	straight-chain alkanes, branched alkanes	OT	dynamic coating	[74]
MIL-101(Fe)	alkanes	OT	dynamic coating	[75]
ZIF-8(G-Z)	branched alkane isomers and aromatic positional isomers	OT	static coating	[76]

### 2.3 MOFs电色谱固定相的分析物类型

#### 2.3.1 手性小分子化合物

与HPLC、GC相比,CEC具有更高的分离效率、更低的固定相用量和成本等优势,因此在新型手性MOFs分离介质的研究中得到了广泛应用。Sun等^[77]^利用原位生长原理成功制备了HKUST-1涂层柱,协同缓冲液中添加的羧甲基*β*-环糊精(CM-*β*-CD)手性选择剂,成功实现了多种手性药物的CEC手性拆分。与未修饰毛细管柱相比,HKUST-1涂层柱可显著增强CM-*β*-CD的手性选择性。Wang等^[78]^基于硅烷偶联原理,将铁基*γ*-CD构建的手性MOFs晶体共价键合到毛细管内壁,并成功将其应用于14种手性药物和1种手性醛的CEC手性分离。Gao等^[79]^基于巯基-烯点击反应原理将L-Cys-PCN-224手性MOFs晶体键合在烯基改性毛细管内壁上,所合成的L-Cys-PCN-224涂层柱成功用于17种对映体化合物的CEC手性拆分。Ding等^[80]^基于原位生长原理制备了ZIF-90涂层毛细管柱,并结合流动相中的乳酸手性选择剂,实现了普萘洛尔、美托洛尔、阿替洛尔、比索洛尔、索他洛尔等手性药物的CEC手性拆分。这些研究结果表明,手性MOFs分离介质与CEC的联用具有广阔的应用前景。

#### 2.3.2 生物大分子

生物大分子作为生物体的重要组成成分,在很多重要的细胞活动中发挥着关键功能。高纯度的生物大分子在药物研发、临床诊断和生物医学等领域具有至关重要的价值。因此,生物大分子的分离分析已经成为新兴的研究热点之一。Geng等^[81]^利用静电相互作用将ZIF-8物理涂覆在毛细管内壁,用作OT-CEC固定相。在最佳分离条件下,该ZIF-8涂层毛细管柱能够在10 min内实现对溶菌酶、细胞色素C、牛血清白蛋白和核糖核酸酶A的快速基线分离。此外,该新型CEC分离分析方法在手性生物分子和蛋清蛋白的分离方面也表现出良好的效果。本研究组采用硅酸钠辅助涂层方法成功地将微孔-介孔多级孔结构的NHP-UiO-66晶体固定在毛细管柱内壁。除了能高效分离多种小分子化合物外,该NHP-UiO-66涂层CEC固定相还对多肽和碱性蛋白质等生物大分子具有出色的分离性能^[82]^。尽管MOFs材料在生物大分子的CEC分离分析领域已经显示出一定的应用潜力,但仍需开展更多深入的研究,以拓展其在蛋白质组学、基因组学以及其他生物医药领域中的实际应用价值。

#### 2.3.3 非手性小分子化合物

MOFs材料作为CEC固定相已被广泛应用于非手性小分子化合物的高效分离分析。本课题组^[83]^利用PDA将BSA固定于DUT-5涂层毛细管柱上,研究了BSA与黄酮类化合物、氟喹诺酮类药物的相互作用。在最佳CE条件下,成功实现了对3种黄酮类化合物和3种氟喹诺酮类化合物的高效基线分离。Li等^[84]^采用原位生长法制备了Bio-MOF-1改性CEC柱,对非甾体类抗炎药、磺胺类药物和氯苯等小分子化合物表现出良好的分离选择性,并展示出优异的稳定性和重复性。此外,本研究组还研究了MOFs材料用作CEC分离介质和固定化酶反应器(IMER)载体材料的潜在应用。通过静电相互作用将乙酰胆碱酯酶(AChE)固定在UiO-66-NH_2_涂层柱上,成功构建了新型的在线酶分析方法^[85]^。与传统共价键合法构建的CE-IMER相比,UiO-66-NH_2_-CEC-IMER不仅具有出色的CEC分离性能,还显著提高了酶的负载量、稳定性和酶分析性能。表3总结了MOFs电色谱固定相的主要类型及分析对象。

**表 3:** MOFs固定相在CEC中的典型应用

Material	Analytes	Type	Form	Ref.
HKUST-1@capillary	propranolol, esmolol, amlodiene	OT-CEC	in situ growth (LPE)	[77]
Fe-CD-MOF@IPTS	anisodamine, pseudoephedrine, synephrine, promethazine	OT-CEC	covalently bonding	[78]
L-Cys-PCN-222	amino acids, fluoroquinolones and other 17 enantiomers	OT-CEC	covalently bonding	[79]
NHP-UiO-66	organic small molecule, peptides, basic proteins	OT-CEC	physical coating (sodium silicate adhesion)	[82]
DUT-5	flavonoids and fluoroquinolones	OT-CEC	physical coating (PDA adhesion)	[83]
Bio-MOF-1	NASIDs, chlorobenzene and other small biomolecules	OT-CEC	in situ growth	[84]
ZIF-8 (cZIF)	polycyclic aromatic hydrocarbons, NASIDs	MC-CEC	covalently bonding	[86]
HKUST-1	polycyclic aromatic hydrocarbons, phenols	OT-CEC	in situ growth (LBL)	[87]
MOF-5	alkyl benzenes, aromatic acids, anilines	OT-CEC	in situ growth (LPE)	[88]
MOF-180	alkyl benzenes, aromatic acids, anilines, chlorobenzenes	OT-CEC	in situ growth (LPE)	[89]
ZIF-90	xylene, dichlorobenzene, chlorotoluene, NASIDs, aniline	OT-CEC	covalently bonding	[90]
JLU-Liu23	epinephrine, isoprenaline, synephrine, terbutaline	OT-CEC	physical coating (sodium silicate adhesion)	[91]
UiO-66-NH_2_	chlorobenzenes, phenoxyacids, phenols	OT-CEC	in situ growth (LPE)	[92]
ZIF-8	hydroquinone, resorcinol, catechol, hydroquinone, resorcinol	OT-CEC	physical coating (PDA adhesion)	[93]
ZIF-8	monoamine compounds	OT-CEC	physical coating (thermocuring)	[94]

NSAIDs: nonsteroidal antiinflammatory drugs; MC-CEC: monolithic columns capillary electrochromatography; LPE: liquid-phase epitaxy; LBL: layer-by-layer.

## 3 总结与展望

综上所述,MOFs涂层作为高性能色谱分离固定相的应用价值备受关注。然而,迄今为止,MOFs色谱固定相的构建方法尚不够完善,仍需要进一步深入的研究。采用填充法制备MOFs色谱固定相存在诸多缺点,包括填料用量大、成本高、柱效低以及重现性差。通过物理涂覆等方法将MOFs固定于硅球表面或毛细管柱内壁,可以有效降低成本且操作较为简便,但涂层的稳定性较差且使用寿命较短。采用共价键合、逐层沉积等方法构建MOFs色谱固定相可以显著提高涂层的稳定性,但制备过程通常较为繁琐且耗时,需要对固定相基底进行多步改性。利用原位合成策略构建MOFs色谱固定相可以进一步提升涂层的稳定性和覆盖率,但合成的MOFs材料的结晶性和多孔性易受制备条件的影响,从而导致涂层的重现性较差。

尽管MOFs的种类繁多,但相当数量的MOFs材料溶剂稳定性较差,容易造成其晶体结构在水或其他溶剂中发生坍塌等问题。受高稳定性MOFs类型的限制,在HPLC和CEC固定相的研究中,研究者们往往更倾向于选择一小部分具有较高水稳定性的MOFs材料(如UiO-66和MIL系列)作为固定相。这在一定程度上限制了MOFs在色谱固定相领域的广泛应用。因此,有必要进一步开发具有更强溶剂稳定性与功能多样性的新型MOFs色谱固定相。

除此之外,目前MOFs色谱固定相的相关研究多数仅聚焦于新型MOFs材料的引入及其分离性能验证,未针对MOFs固定相的理性构建与分离机制两个方面进行充分探讨。因此,为进一步拓展MOFs在色谱分离中的应用潜力,未来在以下几个研究方向还需更多的理论阐释和实际探究:(1)目前已开发的MOFs色谱固定相大多局限于微孔结构(孔径<2 nm),这限制了客体分子在MOFs骨架内的传质效率,从而限制了色谱分离性能。为满足对复杂样品高效分离分析的需求,须进一步开发具有强分离选择性和传质性能的新型MOFs色谱固定相;(2)色谱分离效能与MOFs固定相的骨架结构和制备方法密切相关。因此,在保持MOFs高结晶性和多孔性的基础上,开发普适性强且涂覆质量良好的新型MOFs固定相制备方法对提升其色谱分离性能具有重要意义;(3)耐水功能化MOFs材料及水稳定MOFs固定相制备方法的研究有待拓展,以提高MOFs色谱固定相的寿命,并进一步丰富高实用性MOFs色谱分离介质的种类;(4)MOFs固定相的色谱分离机理有待深入探究,以为构建分离性能更加优异的色谱分离分析方法提供理论指导。综上所述,尽管MOFs材料在色谱分离中的研究仍面临一些困难和挑战,但随着研究者对其结构与功能的持续探索,MOFs材料作为色谱固定相的应用价值有望得到进一步拓展。
